# Genome-Wide Analyses of Exonic Copy Number Variants in a Family-Based Study Point to Novel Autism Susceptibility Genes

**DOI:** 10.1371/journal.pgen.1000536

**Published:** 2009-06-26

**Authors:** Maja Bucan, Brett S. Abrahams, Kai Wang, Joseph T. Glessner, Edward I. Herman, Lisa I. Sonnenblick, Ana I. Alvarez Retuerto, Marcin Imielinski, Dexter Hadley, Jonathan P. Bradfield, Cecilia Kim, Nicole B. Gidaya, Ingrid Lindquist, Ted Hutman, Marian Sigman, Vlad Kustanovich, Clara M. Lajonchere, Andrew Singleton, Junhyong Kim, Thomas H. Wassink, William M. McMahon, Thomas Owley, John A. Sweeney, Hilary Coon, John I. Nurnberger, Mingyao Li, Rita M. Cantor, Nancy J. Minshew, James S. Sutcliffe, Edwin H. Cook, Geraldine Dawson, Joseph D. Buxbaum, Struan F. A. Grant, Gerard D. Schellenberg, Daniel H. Geschwind, Hakon Hakonarson

**Affiliations:** 1 Autism Genetic Resource Exchange, Autism Speaks, Los Angeles, California, United States of America; 2 Department of Genetics, University of Pennsylvania, Philadelphia, Pennsylvania, United States of America; 3 Penn Center for Bioinformatics, University of Pennsylvania, Philadelphia, Pennsylvania, United States of America; 4 Department of Neurology, University of California Los Angeles, Los Angeles, California, United States of America; 5 Center for Applied Genomics, The Children's Hospital of Philadelphia, Philadelphia, Pennsylvania, United States of America; 6 Department of Psychiatry, University of California Los Angeles, Philadelphia, Pennsylvania, United States of America; 7 Center for Autism Research, Semel Institute for Neuroscience and Behavior, University of California Los Angeles, Los Angeles, California, United States of America; 8 Department of Biology, University of Pennsylvania, Philadelphia, Pennsylvania, United States of America; 9 Department of Biomedical Engineering, University of Southern California, Los Angeles, California, United States of America; 10 Porter Neuroscience Research Center, National Institute on Aging, National Institute of Health, Bethesda, Maryland, United States of America; 11 Department of Psychiatry, University of Iowa, Iowa City, Iowa, United States of America; 12 Department of Psychiatry, University of Utah, Salt Lake City, Utah, United States of America; 13 Institute for Juvenile Research, Department of Psychiatry, University of Illinois at Chicago, Chicago, Illinois, United States of America; 14 Department of Psychiatry, Indiana University, Indianapolis, Indiana, United States of America; 15 Department of Biostatistics, University of Pennsylvania, Philadelphia, Pennsylvania, United States of America; 16 Department of Human Genetics, University of California Los Angeles, Los Angeles, California, United States of America; 17 Department of Psychiatry and Neurology, University of Pittsburgh, Pittsburgh, Pennsylvania, United States of America; 18 Center for Molecular Neuroscience and Vanderbilt Kennedy Center, Vanderbilt University, Vanderbilt University, Nashville, Tennessee, United States of America; 19 Department of Psychiatry, University of North Carolina, Chapel Hill, North Carolina, United States of America; 20 Seaver Autism Center for Research and Treatment, Departments of Psychiatry, Neuroscience, Genetics and Genomic Sciences, Mount Sinai School of Medicine, New York, New York; 21 Department of Pediatrics, University of Pennsylvania, Philadelphia, Pennsylvania, United States of America; 22 Department of Pathology and Laboratory Medicine, University of Pennsylvania, Philadelphia, Pennsylvania, United States of America; The University of Queensland, Australia

## Abstract

The genetics underlying the autism spectrum disorders (ASDs) is complex and remains poorly understood. Previous work has demonstrated an important role for structural variation in a subset of cases, but has lacked the resolution necessary to move beyond detection of large regions of potential interest to identification of individual genes. To pinpoint genes likely to contribute to ASD etiology, we performed high density genotyping in 912 multiplex families from the Autism Genetics Resource Exchange (AGRE) collection and contrasted results to those obtained for 1,488 healthy controls. Through prioritization of exonic deletions (eDels), exonic duplications (eDups), and whole gene duplication events (gDups), we identified more than 150 loci harboring rare variants in multiple unrelated probands, but no controls. Importantly, 27 of these were confirmed on examination of an independent replication cohort comprised of 859 cases and an additional 1,051 controls. Rare variants at known loci, including exonic deletions at *NRXN1* and whole gene duplications encompassing *UBE3A* and several other genes in the 15q11–q13 region, were observed in the course of these analyses. Strong support was likewise observed for previously unreported genes such as *BZRAP1*, an adaptor molecule known to regulate synaptic transmission, with eDels or eDups observed in twelve unrelated cases but no controls (*p* = 2.3×10^−5^). Less is known about *MDGA2*, likewise observed to be case-specific (*p* = 1.3×10^−4^). But, it is notable that the encoded protein shows an unexpectedly high similarity to Contactin 4 (BLAST E-value = 3×10^−39^), which has also been linked to disease. That hundreds of distinct rare variants were each seen only once further highlights complexity in the ASDs and points to the continued need for larger cohorts.

## Author Summary

Autism spectrum disorders (ASDs) are common neurodevelopmental syndromes with a strong genetic component. ASDs are characterized by disturbances in social behavior, impaired verbal and nonverbal communication, as well as repetitive behaviors and/or a restricted range of interests. To identify genes likely to contribute to ASD etiology, we performed high density genotyping in 912 multiplex families from the Autism Genetics Resource Exchange (AGRE) collection and contrasted results to those obtained for 1,488 healthy controls. To enrich for variants most likely to interfere with gene function, we restricted our analyses to deletions and gains encompassing exons. Of the many genomic regions highlighted, 27 were seen to harbor rare variants in cases and not controls, both in the first phase of our analysis, and also in an independent replication cohort comprised of 859 cases and 1,051 controls. More work in a larger number of individuals will be required to determine which of the rare alleles highlighted here are indeed related to the ASDs and how they act to shape risk.

## Introduction

The Autism spectrum disorders (ASDs, MIM: 209850) are a heterogeneous group of childhood diseases characterized by abnormalities in social behavior and communication, as well as patterns of restricted and repetitive behaviors [1]. Twin studies have demonstrated much higher concordance rates of ASD in monozygotic twins (92%) than dizygotic twins (10%) [2],[3], indicating a strong genetic basis for autism susceptibility. Although previous work has implicated numerous genomic regions of interest [4]–[8], the identification of specific genetic variants that contribute to ASD risk remains challenging.

Substantial progress towards the identification of genetic risk variants has come from recent characterization of structural variation (*i.e.*, copy number variation or CNV). For example, an initial report involving patients with syndromic autism characterized genomic variation using array comparative genomic hybridization (CGH) and identified large *de novo* CNVs in 28% of cases [9]. Similarly, subsequent work demonstrated that the frequency of *de novo* CNVs is higher in cases versus controls [7],[8]. CNV analyses have proven useful in the identification of regions that are potentially disease-related [8], [10]–[13] and have begun to be employed to advance the candidacy of individual genes, including *NRXN1*, *CNTNAP2*, and *NHE9* [6], [14]–[16]. Recent work characterizing structural variation in cases and ethnically matched controls associating ubiquitin-pathway genes with autism with replicating this finding in the AGRE dataset is likewise notable [17], although family data was not reported here. Using the AGRE dataset as a discovery cohort, along with family information available for AGRE samples, we describe distinct and complementary analyses, prioritizing exonic events over CNVs in introns and intergenic intervals, which provide important new insights into the genetic architecture of the ASDs.

Towards the identification of additional genes and regions that may modulate disease risk, we have assembled a resource characterizing genome-wide structural variation from over nine hundred multiplex ASD families. Presented below are results from analyses contrasting events observed in cases and healthy ethnically matched controls, focusing on three classes of genic events: exonic deletions (eDels), exonic duplications (eDups), and whole gene duplication (gDups). Recovery of known ASD loci – together with the identification of novel regions harboring variants in multiple cases but no controls – supports the utility of this dataset. Consistent with enormous inter-individual variation, we further document a large number of events observed in only individual cases (Table S4). Importantly, all of these data have been made available to the scientific community pre-publication (www.agre.org), greatly enhancing the utility of existing publicly accessible biomaterials and phenotype data. These data further highlight the extent of structural variation in both human and the ASDs and offer an important resource for hypothesis-generation and interrogation of individual loci.

## Results/Discussion

To characterize structural variation in ASD multiplex families and unrelated controls, we typed individuals at 561,466 SNP markers using Illumina HumanHap550 version 3 arrays. After excluding samples that failed to meet QC thresholds (see Table S1), we obtained array data on 3832 individuals from 912 multiplex families enrolled in the Autism Genetic Resource Exchange (AGRE) [18], 1070 disease-free children from the Children's Hospital of Philadelphia (CHOP), and 418 neurologically normal adults and seniors from the National Institute of Neurological Disorders and Stroke (NINDS) control collection [19]. Using the PennCNV software [20], we detected CNVs with a mean size of 59.9 Kb and mean frequency of 24.3 events per individual (see Table S2). Sensitivity compares favorably with previous BAC array-based [9],[21] and SNP-based methods [8], in which mean resolution was observed to be in the range of Mbs and hundreds of Kbs, respectively.

**Figure 1:**
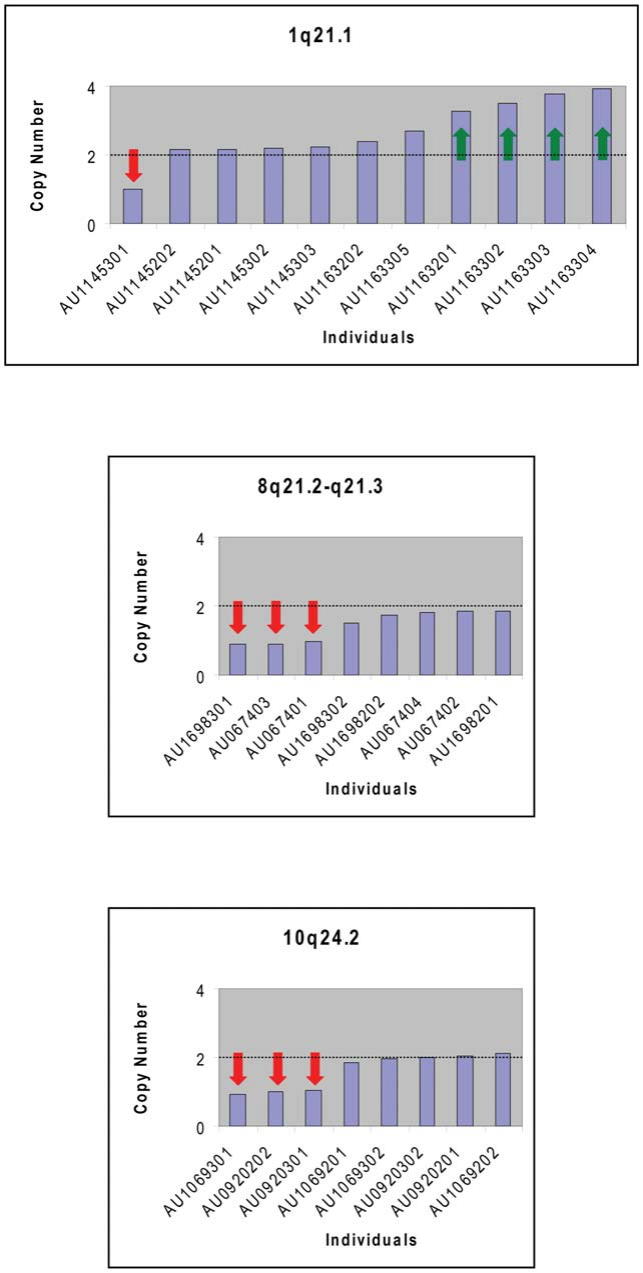
TaqMan experiments validate large de novo CNV calls. To validate results using an independent method we designed TaqMan assays to evaluate gene dosage. Results from representative experiments highlight results at loci at 1q21, 8q21, and 10q24. AGRE individual harboring deletions (red arrows) or gains (green arrows) are indicated.

As a first step towards validation of genotyping accuracy we examined the inheritance of CNVs in the AGRE cohort. Consistent with high quality, 96.2% of CNV calls made in children were also detected in a parent. To explore the issue of genotyping accuracy further, we generated CNV calls for an independently generated data set in which an overlapping set of 2,518 AGRE samples were genotyped using the Affymetrix 5.0 platform [11]. For CNVs (>500 kb) in known ASD regions (e.g. 15q11–13, 16p11.2, and 22q11.21; Table 1) [8],[11],[21],[22], we observed 100% correspondence between the two platforms for individuals genotyped on both platforms. For further confirmation of CNV calls, we compared *de novo* variants identified here to those highlighted in previous analyses of AGRE families. We identified all five *de novo* CNVs reported by Sebat *et al* [7], three of the five *de novo* CNVs reported by Szatmari *et al* [6], one *de novo* CNV within *A2BP1* reported by Martin *et al* [23], and all five 16p11.2 *de novo* deletions reported by Weiss *et al* [11] and Kumar *et al* [10]. Of the two of thirteen *de novo* CNVs reported by Szatmari *et al* not detected as *de novo* in our study, one was very small (2 SNPs, 180 bp on 8p23.2), and the second clearly appears to be inherited (469 SNPs, 1.4 Mb on 17p12). Thus, our data are concordant with several other studies, and provide a more comprehensive picture of *de novo* CNVs in multiplex autism families. To further evaluate the quality of these data on another independent platform, we used Taqman to determine relative copy number at 12 previously unreported *de novo* CNVs identified in AGRE probands, confirming 11/12 loci (Figure 1 and Table S3). Together these results suggest that the CNVs calls we report are consistent and reliable.

**Table 1:** CNVs (>500 kb) on 16p11, 15q11–13, and 22q11 are present in a subset of AGRE families.

Region	#SNP	Length (bp)	Type	AGRE ID	Scored status	Inheritance status	Shared by affected sibling?	Previous reports
15q11–13	1246	5,902,313	dup	AU010601		parent		[22]
15q11–13	1246	5,902,313	dup	AU010604	Autism	Inherited	No	[22]
15q11–13	1246	5,902,313	dup	AU1331202		parent		
15q11–13	1246	5,902,313	dup	AU1331302	Autism	inherited	Yes	
15q11–13	1246	5,902,313	dup	AU1331303	Autism	inherited	Yes	
15q11–13	1130	5,008,629	dup	AU006501		parent		
15q11–13	1130	5,008,629	dup	AU006503	Spectrum	inherited	Yes	AGRE cytogenetic annotation
15q11–13	1130	5,008,629	dup	AU006504	Autism	inherited	Yes	AGRE cytogenetic annotation, [21]
15q11–13	1130	5,008,629	dup	AU1135202	Autism	*de novo*	NA	
15q11–13	1127	4,993,869	dup	AU023303	Spectrum	NA	Yes	[22]
15q11–13	1127	4,993,869	dup	AU023304	Autism	NA	Yes	[21],[22]
15q11–13	1127	4,993,869	dup	AU1607307	Autism	*de novo*	No	
15q11–13	569	3,540,078	del	AU1024202		parent		
15q11–13	569	3,540,078	del	AU1024301	Autism	inherited	NA	
15q11–13	437	1,347,744	dup	AU038504	Autism	*de novo*	No	[22]
15q11–13	287	1,578,642	dup	AU1208301	Autism	*de novo*	No	
15q11–13	273	1,517,841	dup	AU1875202		parent		
15q11–13	98	572,462	dup	AU052003	Autism	NA	Yes	[21]
15q11–13	98	572,462	dup	AU052004	Autism	NA	Yes	
16p11.2	47	530,466	del	AU0154302	Autism	*de novo*	Yes	[10],[11]
16p11.2	47	530,466	del	AU0154303	Autism	*de novo*	Yes	[10],[11],[21]
16p11.2	47	530,466	del	AU029803	Autism	*de novo*	No	[10],[11],[21]
16p11.2	47	530,466	del	AU041905	Autism	*de novo*	No	[10],[11],
16p11.2	47	530,466	del	AU0938301	Autism	*de novo*	No	[10],[11],[21]
16p11.2	47	530,466	dup	AU002901		parent		[11]
16p11.2	47	530,466	dup	AU002903	Autism	inherited	Yes	[11]
16p11.2	47	530,466	dup	AU002904	None	inherited		[11]
16p11.2	47	530,466	dup	AU002905	Autism	inherited	Yes	[10],[11]
22q11.21	512	2,534,567	dup	AU001802		parent		[22]
22q11.21	512	2,534,567	dup	AU001804	Autism	inherited	No	[21],[22]
22q11.21	512	2,534,567	dup	AU004903	Autism	*de novo*	No	[21],
22q11.21	335	1,429,207	dup	AU0991301	Autism	NA	No	
22q11.21	177	728,859	dup	AU1334201		parent		
22q11.21	177	728,859	dup	AU1334302	Spectrum	inherited	No	
22q11.21	149	601,423	del	AU1555302	Autism	NA	NA	

We therefore undertook additional analyses to identify specific loci in which structural variants were enriched in cases versus controls. Because the majority of such variants were intronic or intergenic, we sought to prioritize CNVs most likely to interfere with the molecular function of specific genes. We first filtered CNV calls to include only exonic deletions (eDels) observed to overlap with a RefSeq gene. Overall, such eDels were observed at similar frequencies in AGRE cases, 1^st^ degree relatives of AGRE cases, and unrelated controls (CHOP and NINDS cohorts), with an average of ∼2 such variants per person (Table S2). To identify events related to the ASDs we then looked for genes harboring eDels in at least one case but no unrelated controls. Among the 284 genes that met this criteria (Table S4) we observed several known ASD or mental retardation genes including: *ASPM* [24], *DPP10* [8], *CNTNAP2* [25],[26], *PCDH9* [16], and *NRXN1* [6].

**Figure 2:**
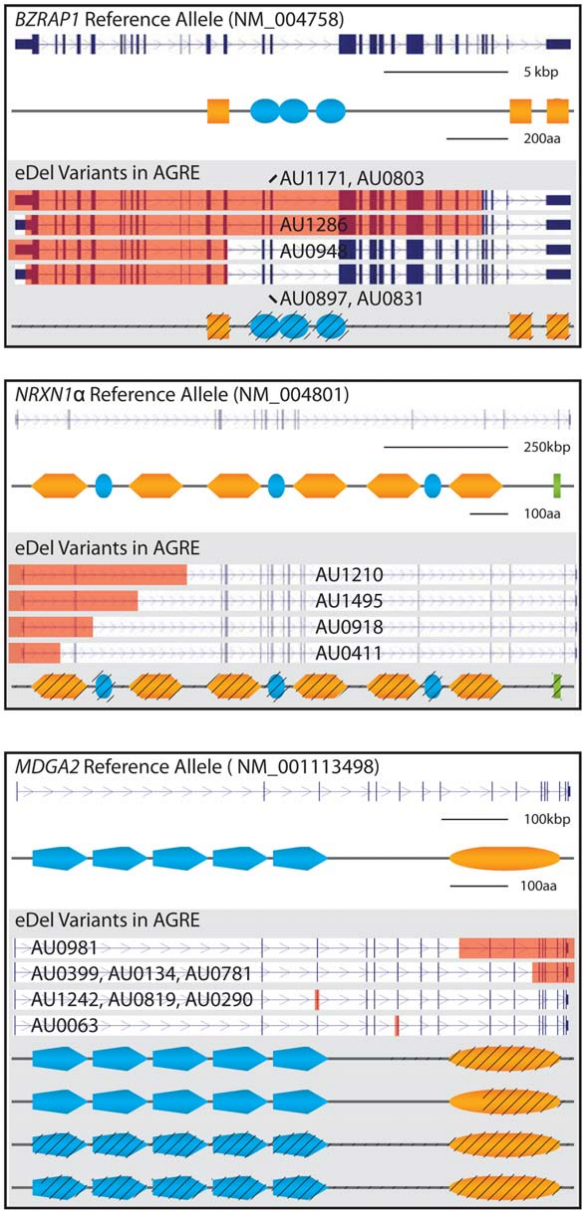
Rare exonic deletions (eDels) in *NRXN1* and novel candidate genes alter predicted protein structures. For each of *BZRAP1* (a) *NRXN1* (b) and *MDGA2* (c) reference loci and encoded proteins (top) are contrasted against mutant loci and corresponding proteins (bottom; grey shading). Unique genomic deletions and corresponding protein truncations are highlighted in red and with black hatching, respectively. Schematized protein domains genes are as follows: *BZRAP1*—Src homology-3 (orange square), Fibronectin, type III (blue oval); *NRXN1*—Laminin G (orange hexagon), EGF-like (blue oval), 4.1 binding motif (green rectangle); *MDGA2*—IG-like domains (blue pentagon), MAM aka Meprin/A5-protein/PTPmu (blue oval).

To enrich for genes most likely to contribute to ASD risk, we used family-based calling to evaluate which of these genes carried eDels in three or more cases from at least two unrelated families (Table S5). This stringent filtering resulted in 72 genes at 55 loci, including *NRXN1*. This is notable, given that eleven distinct disease-linked *NRXN1* variants have been identified [6],[8],[15],[27],[28]. Neurexin family members are known to interact functionally with ASD-related neuroligins [29]–[32], and likewise play an important role in synaptic specification and specialization [33],[34]. eDels in more recently identified candidates, including *DPP10* and *PCDH9*, were likewise retained. Similarly, recovery of *RNF133* and *RNF148* within intron 2 of *CADPS2* [7],[35] highlights additional complexity at this locus. Although CNV breakpoints cannot be mapped precisely using SNP data alone, it is possible to determine overlap with protein coding exons and use these data to predict impact on gene function. Consistent with perturbation of function, distinct alleles at the loci highlighted here are predicted to eliminate or truncated the corresponding protein products (Figure 2).

Importantly, CNVs at a majority of these eDel loci show unique breakpoints in different families and/or result in the loss of distinct exons, demonstrating that they are independent. Moreover, because it is well established that CNVs at a subset of loci show identical breakpoints in unrelated individuals [10], this result is likely to underestimate the extent to which variants described here arose independently. Results from multi-dimensional scaling are likewise consistent with the interpretation that variants we highlight arose independently (Figure S1).

Given the large number of variants identified, it was critically important to confirm in an independent case-control analysis, how many of these eDels were truly overrepresented in cases, as opposed to being potentially attributable to Type I error. To address this concern, we sought to determine eDel frequency in these same genes in a replication dataset comprising 859 independently ascertained ASD cases and 1051 unrelated control subjects from the Autism Case Control cohort (ACC, see Description in Methods). One third of the loci identified in the discovery phase were observed in one or more ACC controls (18/55; 32.7%), suggesting that while rare, eDels at these loci are not limited to ASD cases and family members. In contrast, and providing evidence for formal replication, 14 separate loci encompassing 22 genes were observed to carry eDels in both AGRE and ACC cases, but none of 2539 controls (Table S2).

**Figure 3:**
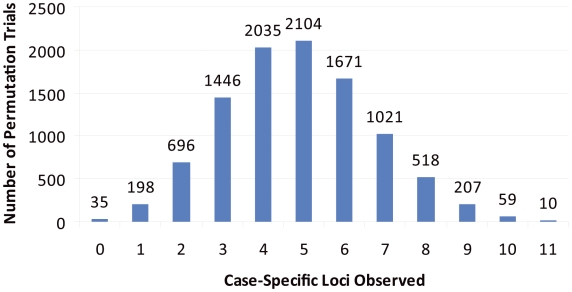
Observed replication unlikely to be attributable to chance alone. We performed 10,000 phenotype permutation trials on replication data and determined for each the number of loci harboring CNVs in cases but not controls. Thus, within each trial, the number of loci absent from controls in the replication cohort was determined. None of the permutation trials generated as many case-specific loci as observed in our actual dataset (n = 14; p<0.0001).

Our replication data lend strong support to the involvement of specific loci in the ASDs (Table 2). However, to ensure that these results were not observed by chance alone, we performed 10,000 permutation trials on data from the replication cohort by permuting case/control status across individuals. In each permuted dataset, we maintained the same numbers of cases and controls as in the original data, and calculated the number of genes harboring CNVs exclusively in cases. None of the 10,000 permutation trials gave results comparable to experimental observations for replicated case-specific loci (n = 14; p<0.0001; Figure 3). In contrast, findings comparable to those for non-replicated loci (highlighted as case-specific in the discovery phase but subsequently seen in replication controls) were seen in controls in 246/10,000 trials (n = 18; p = 0.02; Figure S2). Although additional experimental work in independent cohorts will be required to determine if variation in any of the genes highlighted here do in fact impact ASD risk, no more than 5 replicated loci would be predicted to be observed by chance alone.

**Table 2:** A subset of eDel loci were observed to harbor rare variants in both discovery and replication cohorts, but none of 2539 controls. eDel: exonic deletion; ACRD: autism chromosome rearrangement database (http://projects.tcag.ca/autism/).

Gene	Locus^A^	Unrelated Discovery Cases (n = 912)	UnrelatedDiscovery Controls (n = 1488)	Unaffected Carrier Fraction^B^	Unrelated Replication cases (n = 859)	Unrelated Replication controls (n = 1051)	ACRD?	Combined P-value (Fisher Exact)
*- CA6*	1p36	3	0	0.50	1	0	No	0.028
*- OR1C1*	1q44	3	0	0.40	1	0	No	0.028
*- NRXN1*	2p16	5	0	0.46	4	0	Yes	3.3×10^−4^
*- GALNT13*	2q23.3	4	0	0.54	1	0	Yes	0.012
*- SUCLG2*	3p14	2	0	0.25	1	0	Yes	0.069
*- KIAA1586*	6p12	3	0	0.36	2	0	No	0.012
*- RNF133*	7q31.3	3	0	0.60	1	0	Yes	0.028
*- RNF148*								
*- SSSCA1*	11q13	3	0	0.43	1	0	No	0.028
*- FAM89B*								
*- UNC93B1*	11q13	3	0	0.25	1	0	No	0.028
*- MDGA2*	14q21.3	8	0	0.56	2	0	No	1.4×10^−4^
*- LOC650137* C	15q11.2	26	0	0.64	2	0	Yes	1.3×10^−11^
*- OR4M2*								
*- OR4N4*								
*- FLYWCH1* D	16p13	3	0	0.40	3	0	No	4.8×10^−3^
*- KREMEN2*								
*- PAQR4*								
*- PKMYT1*								
*- BZRAP1* E	17q22	6	0	0.50	2	0	No	8.0×10^−4^
*- C19orf19* F	19p13	3	0	0.40	8	0	No	5.5×10^−5^
*- MADCAM1*								

A Predicted event sizes in bps for unrelated AGRE^*^ and ACC^#^ cases are as follows: ***CA6*** - 2317^*^, 12464^*^, 19146^*^, 19145^#^; ***OR1C1*** - 44644^*^, 577691^*^, 1464677^*^, 44643^#^; ***NRXN1*** - 19979^*^, 152437^*^, 241327^*^, 373015^*^, 439406^*^, 134010^#^, 161199^#^, 256373^#^, 533842^#^; ***GALNT13*** - 14126^*^, 46413^*^, 113282^*^, 24100^#^; ***SUCLG2*** - 2192^*^, 1389749^*^, 1389748^#^; ***KIAA1586*** - 36354^*^, 36902^*^,157321^*^, 36901^#^, 67160^#^; ***RNF133/RNF148*** - 33473^*^, 37226^*^, 1515817^*^, 43966^#^; ***SSSCA1/FAM89B*** – 21993^*^, 21993^*^, 21993^*^, 123569^#^; ***UNC93B1*** - 11410^*^, 19223^*^, 84727^*^, 159861^#^; ***MDGA2*** – 19651^*^, 23292^*^, 57714^*^, 58528^*^, 122985^*^, 131623^*^, 150178^*^, 226468^*^, 194601^#^, 288518^#^; ***LOC650137/OR4M2/OR4N4*** – 591007^*^ in 26 families, 24941^#^ and 926360^#^; ***FLYWCH1/KREMEN2/PAQR4/PKMYT1*** - 40468^*^, 81127^*^, 88373^*^, 82786^#^, 82786^#^, 124947^#^; ***BZRAP1*** – 10102^*^, 10102^*^, 16897^*^, 18532^*^, 22806^*^, 34235^*^, 29600^#^, 28360^#^; ***C19orf19*** **/** ***MADCAM1*** - 100187^*^, 171989^*^, 187147^*^, 98264^#^, 103788^#^, 277715^#^, 280201^#^, 292525^#^, 294446^#^, 344224^#^, 384324^#^.

B For eDels at a given locus, the ratio of *unaffected* carriers (siblings or parents) to total number of carriers (cases and family members).

C The significant difference in CNV frequency between AGRE and ACC cases (p = 2.6×10^−6^), along with multiple instances of similar variation in the DGV (see Tables S4 and Table S5), suggests that additional factors – including some potentially unrelated to diagnosis – may be relevant here. Sparse SNP coverage along with regional complexity (large segmental duplications) is also likely to increase false positive and false negatives at this locus. Replication data (and corresponding *p* Value) is for *OR4N4*, as only one eDel at either *LOC650137* or *OR4M2* was observed amongst ACC cases.

D A comparable number of eDels were observed at multiple neighboring genes; carrier fraction corresponds to *FLYWCH1*, the lowest observed at this locus.

E Joint consideration of eDels (n = 8) and eDups (n = 4) at *BZRAP1* further improves statistical support for this locus (*p* = 2.3×10^−5^).

F Note extreme telomeric position of this locus which may undermine/interfere with reliable calling of structural variants. CNV counts and carrier fraction corresponds to *MADCAM1*; fewer variants were observed at *C19orf19* amongst ACC cases and carrier fraction was higher than that for *MADCAM1*.

Despite the challenges associated with obtaining statistical support for individually rare events [7],[36] we next sought to assign *P* values for replicated eDel loci. We were able to obtain support for each of the following loci: *BZRAP1* at 17q22 (*p* = 8.0×10^−4^), *NRXN1* at 2p16.3 (*p* = 3.3×10^−4^), *MDGA2* at 14q21.3 (*p* = 1.3×10^−4^), *MADCAM1* at 19q13 (*p* = 5.5×10^−5^), and a three gene locus at 15q11 (*p* = 1.3×10^−11^). CNV calls at each of 15q11 and 19p13 are highly-error prone, suggesting that results here be interpreted with caution (see footnotes C and F in Table 2). Recovery of *NRXN1*, however, provides confidence for involvement of additional loci that were likewise replicated. Benzodiazapine receptor (peripheral) associated protein 1 (*BZRAP1*, alternatively referred to as *RIMBP1*), is an adaptor molecule thought to regulate synaptic transmission by linking vesicular release machinery to voltage gated Ca2+ channels [37]. Identification of this synaptic component here, in a hypothesis-free manner, is particularly satisfying and also provides additional support for synaptic dysfunction in the ASDs [29],[38]. Less is known about *MDGA2* [39], although comparison of the predicted protein to all others within GenBank by BLASTP indicated an unexpectedly high similarity to Contactin 4 (24% identity over more than 500 amino acids; Expect = 3×10^−39^). Given previous reports of hemizygous loss of *CNTN4* in individuals with mental retardation [40] and autism [17],[41]. similarity between MDGA2 and CNTN4, surpassed only by resemblance to MDGA1, is notable. Likewise intriguing in light of the suggestion that common variation in cell adhesion molecules may contribute to autism risk [42] is the structural likeness of MDGA2 to members of this family of molecules.

Although some published analyses emphasize the greater contribution of gene deletion events in autism pathogenesis [7], there are also clear examples of duplications that strongly modulate ASD risk [43],[44]. We therefore conducted a parallel analysis of duplications, distinguishing between events involving entire genes (gDups) which might increase dosage and those restricted to internal exons (eDups) which could give rise to a frameshift or map to a chromosomal region distinct from the reference gene. For gDups, we identified 449 genes that were duplicated in at least one AGRE case but no CHOP/NINDS controls (Table S4). Of those, 200 genes at an estimated 63 loci, including genes at 15q11.2 [43], met the more stringent criteria of being present in three or more cases from at least two independent families (Table S5). Of these, 11.5% (23/200) were also seen in ACC controls, whereas 24.5% (49/200) were case-specific in the replication cohort. Strong statistical support was obtained for established loci (e.g. *p* = 9.3×10^−6^ for *UBE3A* and other genes in the PWS/AS region at 15q11–q13), and nominal evidence was observed for the following novel loci: *CD8A* at 2p11.2 (*p* = 0.069), *LOC285498* at 4p16.3 (*p* = 0.028), and *CARD9/LOC728489* at 9q34.3 (p = 0.005).

For eDups, we reasoned that duplication of one or more internal exons could serve to disrupt the corresponding open reading frame and be predicted to impair gene function as a result. Despite the caveat that observed copy number gains need not map to the wild-type locus, known ASD genes including *TSC2* [45] and *RAI1* [44],[46] within the Potocki-Lupski Syndrome critical interval were amongst the 159 loci observed in at least one AGRE case, but no CHOP/NINDS controls (Table S4). Such events were also seen in one family at the *NLGN1* locus, which is of interest given previous support for *NLGN3* and *NLGN4* [29]. Filtering of these results, using the more stringent criteria employed above in consideration of eDels, limited this set of events to 76 loci observed in at least three cases from two separate families (Table S5). Interestingly, *BZRAP1*, reported above to harbor eDels at significantly higher frequencies in AGRE and ACC cases versus controls (*p* = 8.0×10^−4^), was amongst these, with eDups observed here in four unrelated AGRE cases (screening *p* = 0.021). Eight other genes, including the voltage gated potassium channel subunit *KCNAB2* (*p* = 4.7×10^−3^) remained absent from ACC controls and were also replicated in the independent case cohort. Although eDups at *BZRAP1* were not detected in ACC cases, eDels at this locus were replicated, underscoring the importance of variation here. When considering eDels and eDups at the *BZRAP1* locus together, the likelihood of such an observation occurring by chance alone is small (*p* = 2.3×10^−5^).

**Figure 4:**
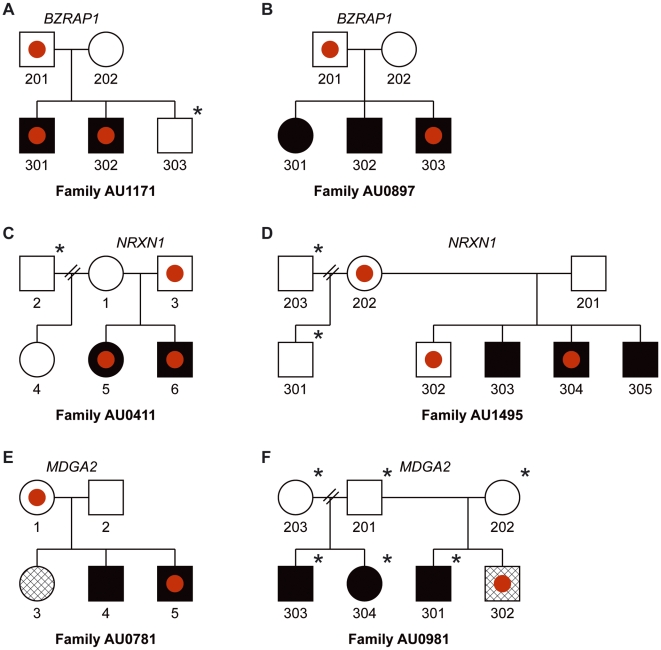
Exonic deletions, although enriched in cases versus controls, show imperfect segregation with disease in multiplex families. Pedigrees for representative AGRE families harboring exonic deletions in *BZRAP1* (A,B), kb), *NRXN1* (C,D), and *MDGA2* (E,F) are illustrated. Red filled circles correspond to exonic deletions. Black stars (upper right) highlight individuals for which CNV calls were not obtained (not genotyped or failing to meet criteria for quality control).

Although none of the variants we highlight were observed in any of 2539 unrelated controls, key events, including eDels at *NRXN1*, *BZRAP1*, and *MDGA2* were observed in both cases and non-autistic family members (Figure 4). This is in keeping with previous work which suggests that haploinsufficiency at *NRXN1* may contribute to the ASDs [15], but is insufficient to cause disease. Such data are also consistent with the well established finding of the “broader autism phenotype”, such as subclinical language and social impairment in first degree relatives of cases with an ASD, which supports a multi-locus model [47],[48]. We were also surprised to see that key variants at these loci appear to be transmitted to only a subset of affected individuals in some families (Figure 4). These observations parallel findings at other major effect loci including 16p11.2 [11] and *DISC1* [49],[50] and are consistent with a model in which multiple variants, common and rare, act in concert to shape clinical presentation [51]–[53]. Results are also consistent with the idea that true risk loci are likely to show incomplete penetrance and imperfect segregation with disease [13], a reality that will complicate gene finding efforts. Related to this is that substantial effort will be required to determine whether rare alleles of moderate effect act independently on distinct aspects of disease (endophenotype model) or together to undermine key processes in brain development (threshold model). How distinct alleles may interact to shape presentation is yet another question that will require larger cohorts along with multigenerational families to resolve [54].

By limiting CNV calls to include only exonic deletions (eDels) and duplications (eDups and gDups), we have attempted to enrich for variants most likely to impact gene function and in doing so improve the signal to noise ratio similar to work in other complex diseases [55]. At the same time, like other gene-based strategies, we preserve our ability to consider eDels involving the same transcriptional unit as separate but equivalent. Given that such events appear rare, this is an important consideration.

Pathway analysis by DAVID [56] found support for overrepresentation of cell adhesion molecules amongst recurrent eDel genes (uncorrected p = 0.002; *CDH17*, *PCDH9*, *LAMA2*, *MADCAM1*, *NRXN1*, *POSTN*, *SPON2*) , although it should be noted that this analysis does not adjust for gene size and may favor larger genes. Nevertheless, aside from *SPON2* no eDels in these genes were observed in any of the controls interrogated. In contrast, no evidence for such overrepresentation was observed for genes in the ubiquitin degradation pathway and neither term was highlighted as overrepresented amongst eDups or gDups. Given that this study focused only on events encompassing RefSeq exons, differences from Glessner and colleagues [17] are to be expected.

Despite the large cohorts interrogated at each phase of our investigations, only a minority of loci (established or novel) were replicated between AGRE and ACC cases. For example, variants at each of the following previously reported loci were observed multiple times in AGRE cases but not once amongst ACC probands: *PCDH10* and *DPP10* (eDels), *RAI* and *TSC2* (eDups), and *DIDO1* (gDups). This suggests that even with current numbers, the present experiments are underpowered to obtain replication for a subset of recurrent variants. Because events seen only in single cases collectively account for a substantial fraction of observed variation even larger cohorts still will be required for a thorough understanding of the genetic basis of complex disorders like the ASDs.

In summary, we have performed a high resolution genome-wide analysis to characterize the genomic landscape of copy number variation in ASDs. Through comparison of structural variation in 1,771 ASD cases and 2,539 controls and prioritization of events encompassing exons we identified more than 150 loci harboring rare variants in multiple probands but no control individuals. For each class of structural variant interrogated, the recovery of known loci serves to validate the methods employed and results obtained. Greatest confidence should be placed in loci harboring variants in multiple unrelated cases but no controls and also recovered in both screening and replication cohorts. Amongst novel genes, best support was obtained for *BZRAP1* and *MDGA2*, intriguing candidate genes for which additional study is warranted.

## Methods

### Sample ascertainment

For initial screening we assembled three sample collections: 1) 943 ASD families (4,444 unique subjects) from the Autism Genetic Resource Exchange (AGRE) collection; 2) 1,070 de-identified and unrelated children of European ancestry from the Children's Hospital of Philadelphia (CHOP), with no evidence of neurological disorders; 3) 542 unrelated neurologically normal adults and seniors of European ancestry from the National Institute of Neurological Disorders and Stroke (NINDS) control collection. The AGRE families include 917 multiplex families, 24 simplex families and 2 families without an ASD diagnosis. For all analyses, AGRE cases annotated with “Autism” (n = 1,463), “Broad Spectrum” (n = 149) or “Not Quite Autism” (n = 71) were treated equally and as affected. Samples from AGRE and NINDS were genotyped using DNA extracted from Epstein-Barr Virus (EBV)-transformed lymphoblastoid cell lines, while the CHOP controls were genotyped using DNA extracted from whole blood. All AGRE and control samples included in these analyses were genotyped on the Illumina HumanHap550 version 3 arrays, and 281 samples genotyped on version 1 arrays were excluded from the present analysis. Since the NINDS controls were genotyped at a different location and time, they were used to assess the frequency of specific CNVs in an independent cohort and to address concerns of cell line artifacts. This study was approved by the Institutional Review Board of Children's Hospital of Philadelphia. All subjects provided written informed consent for the collection of samples and subsequent analysis.

The Autism Case-Control (ACC) cohort included 859 cases from multiple sites within the United States, all of whom were of European ancestry affected with ASD. Of those, 703 were male and 156 were female; 828 met diagnostic criteria for autism, and 31 met criteria for other ASDs. Subjects ranged from 2–21 years of age when the Autism Diagnostic Interview (ADI) was given. Of the case subjects, 54% were from simplex families with the balance coming from multiplex families. The control group used for replication included 1051 children of self-reported Caucasian ancestry who had no history of ASDs. These controls were recruited by CHOP nursing and medical assistant staff under the direction of CHOP clinicians within the CHOP Health Care Network, including four primary care clinics and several group practices and outpatient practices that included well child visits.

### Detection and annotation of copy number variation

For each data set, we applied identical and stringent quality control criteria to remove samples with low signal quality. CNV calls were generated using PennCNV [20], an algorithm which employs multiple sources of information, including total signal intensity, allelic intensity ratios, SNP allele frequencies, distance between neighboring SNPs, and family information to generate calls. We excluded samples meeting any of the following criteria: a) standard deviation for autosomal log R ratio values (LRR_SD) higher than 0.28, b) median B Allele Frequency (BAF_median) higher than 0.55 or lower than 0.45, c) fraction of markers with BAF values between 0.2 and 0.25 or 0.75 and 0.8 (BAF_drift) exceeded 0.002. We also excluded from our analysis CNVs within *IGLC1* (22q11.22), *IGHG1* (14q32.33) and *IGKC* (2p11.2), and the T cell receptor constant chain locus (14q11.2), as well as CNVs in chromosomes showing evidence of heterosomic aberrations (chromosome rearrangements in sub-populations of cells) in BeadStudio.

CNV calls were mapped onto genes by identifying overlap with RefSeq exons, the coordinates of which we obtained from the UCSC table browser. Deletion events overlapping with exons retrieved in this way were listed as eDels. eDups were defined as gains overlapping one or more coding exons and seen to be internal to the beginning and end of the corresponding transcript. Gains observed to encompass all exons for a given gene were annotated as gDups. P values for relative CNV burden in cases and controls were calculated at each locus by Fisher's exact test. P values presented in Table S2, S4, S5 have not been subjected to correction for multiple testing. To compare our CNV calls with other publications that have used AGRE families [10],[11],[21],[22], we examined published calls on the same individuals with the same AGRE identifiers. The CNV calls were retrieved from the Supplementary Materials of each corresponding publication.

### Quantitative PCR for CNV validation

TaqMan primer/probe sets were designed to query random CNVs using FileBuilder 3.0 on the repeat-masked human genome (NCBI_36; March 2006 release; http://genome.ucsc.edu/). For each assay, 10 ng of genomic DNA was assayed in quadruplicate in 10-µL reactions containing 1× final concentration TaqMan Universal Master Mix (ABI part number 4304437), and 200 nM of each primer and probe. Cycling was performed under default conditions in 384-well optical PCR plates on an ABI 7900 machine. Copy number was defined as 2^−ΔΔ*C*T^, where Δ*C*_T_ is the difference in threshold cycles for the sample in question normalized against an endogenous reference (RNAseP) and expressed relative to the average values obtained by three arbitrary control DNAs. A list of TaqMan probes against the 12 CNVs tested is included in Table S3.

## Supporting Information

Figure S1 Multi-dimensional scaling plot of AGRE affected subjects, with red cross highlighting subjects carrying the eDels. Subjects of European ancestry are clustered toward the right side of the triangle.
(0.11 MB DOC)

Figure S2 We performed 10,000 phenotype permutation trials on replication data and determined for each the number of loci harboring CNVs exclusively in controls. During each trial a new set of control-specific loci was identified and the number of these absent from cases determined. We observed results comparable to those obtained experimentally (n = 18) in 246 of 10,000 trials (p = 0.02).
(0.03 MB DOC)

Table S1 Description of AGRE sample used in the analysis.
(0.03 MB DOC)

Table S2 Summary of CNVs in AGRE cases, first-degree relatives, and unrelated controls.
(0.04 MB DOC)

Table S3 TaqMan primers and probes used in CNV validation.
(0.04 MB DOC)

Table S4 Exonic del/dups (Singletons and recurrent).
(0.14 MB XLS)

Table S5 Exonic del/dups (Recurrent in unrelated families).
(0.45 MB XLS)
