# Supplementary material for: Genome-Wide Analyses of Exonic Copy Number Variants in a Family-Based Study Point to Novel Autism Susceptibility Genes
Source: PLoS Genet. 2009 Jun 26;5(6):e1000536. doi: 10.1371/journal.pgen.1000536 (PMC2695001; doi:10.1371/journal.pgen.1000536)
Supplement: Figure S1 — Multi-dimensional scaling plot of AGRE affected subjects, with red cross highlighting subjects carrying the eDels. Subjects of European ancestry are clustered toward the right side of the triangle. (0.11 MB DOC) [file pgen.1000536.s001.doc]

**Supplementary Figure 1.** Multi-dimensional scaling plot of AGRE affected subjects, with red cross highlighting subjects carrying the eDels. Subjects of European ancestry are clustered toward the right side of the triangle.
