# Supplementary material for: Genome-Wide Analyses of Exonic Copy Number Variants in a Family-Based Study Point to Novel Autism Susceptibility Genes
Source: PLoS Genet. 2009 Jun 26;5(6):e1000536. doi: 10.1371/journal.pgen.1000536 (PMC2695001; doi:10.1371/journal.pgen.1000536)
Supplement: Figure S2 — We performed 10,000 phenotype permutation trials on replication data and determined for each the number of loci harboring CNVs exclusively in controls. During each trial a new set of control-specific loci was identified and the number of these absent from cases determined. We observed results comparable to those obtained experimentally (n = 18) in 246 of 10,000 trials (p = 0.02). (0.03 MB DOC) [file pgen.1000536.s002.doc]

**Supplementary Figure 2.** We performed 10,000 phenotype permutation trials on replication data and determined for each the number of loci harboring CNVs exclusively in controls. During each trial a new set of control-specific loci was identified and the number of these absent from cases determined. We observed results comparable to those obtained experimentally (n=18) in 246 of 10000 trials (p=0.02).
