# Supplementary material for: Genome-Wide Analyses of Exonic Copy Number Variants in a Family-Based Study Point to Novel Autism Susceptibility Genes
Source: PLoS Genet. 2009 Jun 26;5(6):e1000536. doi: 10.1371/journal.pgen.1000536 (PMC2695001; doi:10.1371/journal.pgen.1000536)
Supplement: Table S1 — Description of AGRE sample used in the analysis. (0.03 MB DOC) [file pgen.1000536.s003.doc]

**Supplementary Table 1** **–** Description of AGRE sample used in the analysis.

*CHOP Control Cohort:*

1110 samples genotyped

1070 retained after QC (96% pass rate)

*NINDS Control Cohort:*

540 samples genotyped

418 retained after QC (77% pass rate)

*AGRE Family Cohort:*

4163 samples genotyped on v3 arrays

3832 retained after QC (92% pass rate)

*ACC Cases & Controls:*

see Glessner *et al., 2009, Nature* for a full description
