# Supplementary material for: Genome-Wide Analyses of Exonic Copy Number Variants in a Family-Based Study Point to Novel Autism Susceptibility Genes
Source: PLoS Genet. 2009 Jun 26;5(6):e1000536. doi: 10.1371/journal.pgen.1000536 (PMC2695001; doi:10.1371/journal.pgen.1000536)
Supplement: Table S2 — Summary of CNVs in AGRE cases, first-degree relatives, and unrelated controls. (0.04 MB DOC) [file pgen.1000536.s004.doc]

**Supplementary Table 2 –** Summary of CNVs in AGRE cases, first-degree relatives, and unrelated controls.

|  | AGRE affected | AGRE unaffected (siblings/parents) | NINDS controls | CHOP controls |
| --- | --- | --- | --- | --- |
| N= | 1673 | 2159 | 418 | 1070 |
| Mean # CNV | 24.7 | 25.2 | 20.5 | 23.3 |
| Mean # eDels | 2.0 | 2.1 | 2.3 | 2.6 |
| Mean # eDups | 6.0 | 6.3 | 2.2 | 4.2 |
| Mean # gDups | 4.0 | 4.1 | 1.0 | 2.5 |
