# Supplementary material for: Genome-Wide Analyses of Exonic Copy Number Variants in a Family-Based Study Point to Novel Autism Susceptibility Genes
Source: PLoS Genet. 2009 Jun 26;5(6):e1000536. doi: 10.1371/journal.pgen.1000536 (PMC2695001; doi:10.1371/journal.pgen.1000536)
Supplement: Table S3 — TaqMan primers and probes used in CNV validation. (0.04 MB DOC) [file pgen.1000536.s005.doc]

**Supplementary Table 3** **–** TaqMan primers and probes used in CNV validation.

*Reporter and reporter quencher are FAM and NFQ, respectively, unless noted*

**AssaBSA15**

Target = human BCL9

forward primer = CTGAGTTGATTTTTGGTTAAGTTGATTCCTT

reverse primer = GGACCTGAAATTCGAGGATTCTGT

reporter sequence = TAGGAATGGGCATTAATAC

**AssaBSA16**

Target = human NLRP3

forward primer = AGTGCAACCCAGGCTTTCTATTT

reverse primer = GTGTTTCTAACGCACTTTTTGTCTCA

reporter sequence = CAGACAACCTGTAAAAGC

**AssaBSA20**

Target = human NKX3-2

forward primer = TGGAAGCTCTATTCGCTGTATTTTTTCT

reverse primer = CCAAAAGTCGGGAAAAGACAGTTT

reporter sequence = CATGCCCTCCTGGACGC

**AssaBSA21**

Target = human HHIP-itg

forward primer = TCATCTCAGTTGTGATCGTTCTGTTTT

reverse primer = AGGGTGTGCAGAAATGGTACTTAATT

reporter sequence = TCTACATCGTGAAATTAC

**AssaBSA22**

Target = human 4q32.1

forward primer = TGAGTAACAGCATTTATCATGGCTTGA

reverse primer = GGAAAAGGTTTTGAAAACATTGTTATCACAGT

reporter sequence = CCTAAGATCAGGCAATTAG

**AssaBSA23**

Target = human 6q16.1

forward primer = AGTGACAGTACATGCAACAGTTCAT

reverse primer = GCTCCTCTGTAGCTGTCAGTTC

reporter sequence = CTGTGCCAAACTTCA

**AssaBSA25**

Target = human 8q21.2

forward primer = AGTGTAGGTGCAATCAAAGAGAATGA

reverse primer = CTCAATTGTTTTAAAATATTGGGCAAAGTTCA

reporter sequence = ATAAGTGGTTTAGCATTTCTG

**Supplementary Table 3** cont. **–** TaqMan primers used in CNV validation.

*Reporter and reporter quencher are FAM and NFQ, respectively, unless noted*

**AssaBSA26**

Target = human HPSE2-in

forward primer = TCAGTGAGGTCTGGGTTCAATATCT

reverse primer = TGCTGCTCATATGTTATCAAAGCATTATATCA

reporter sequence = TTGGCTGTCCGCCTTGT

**AssaBSA27**

Target = human TAT

forward primer = GCTTCTTGGAGGCTGCTTTCT

reverse primer = CACCACTGCCTGATCAGCTT

reporter sequence = TTGGAAGGTAAAAATCTC

**AssaBSA28**

Target = human PPP1R16B

forward primer = CCAGCTGGTAATGTTGTCCTTCT

reverse primer = GAGAGTAGCACGGGCTTCT

reporter sequence = CACTCGCAGAACCCCA

**AssaBSA29**

Target = human BHLHB4

forward primer = GCGTAGCCGTGGCTTAGT

reverse primer = CCATGGCCGAGCTCAAGT

reporter sequence = CAGGTACGCGTCCCC

**AssaBSA30**

Target = human DMD

forward primer = GATGGACTTCTTATCTGGATAGGTGGTA

reverse primer = GAGTCTCAAATATAGAAACCAAAAATTGATGTGT

reporter sequence = CAACATCTGTAAGCACATTAA

**AssaBSA32**

Target = RNaseP endogenous control

reporter = VIC; quencher = TAMRA; primer limited

Part Number 4316844 (applied biosystems)
